# Clinical correlates and short-term prognostic value of CT-detected subclinical pulmonary congestion in acute STEMI without overt heart failure

**DOI:** 10.3389/fmed.2026.1927125

**Published:** 2026-09-08

**Authors:** Minghui Hua, Rong Liang, Yufan Gao, Keyi Cui, Jiwei Sun, Shuo Liang, Ximing Li, Hong Zhang

**Affiliations:** 1 Department of Radiology, Chest Hospital, Tianjin University, Tianjin, China; 2 Department of Breast Imaging, Tianjin Medical University Cancer Institute and Hospital, National Clinical Research Center for Cancer, Tianjin, China; 3 Academy of Medical Engineering and Translational Medicine, Tianjin University, Tianjin, China; 4 Department of Cardiology, Chest Hospital, Tianjin University, Tianjin, China

**Keywords:** computed tomography, early risk stratification, prognosis, ST-segment elevation myocardial infarction, subclinical pulmonary congestion

## Abstract

**Background:**

Subclinical pulmonary congestion may occur after ST-segment elevation myocardial infarction (STEMI) before overt heart failure (HF) develops. However, its clinical significance remains unclear. This study aimed to investigate the clinical correlates and in-hospital prognostic significance of computed tomography (CT)-detected subclinical pulmonary congestion in acute STEMI patients without overt HF.

**Methods:**

We retrospectively enrolled 276 consecutive patients with acute STEMI classified as Killip class I who underwent chest CT at admission. Quantitative CT-derived mean lung density was used to assess subclinical pulmonary congestion. Patients were stratified into subgroups using K-means clustering based on mean lung density. A logistic regression model was constructed to discriminate the two subgroups, and the importance of clinical features was ranked accordingly. For prognostic analysis, univariable and multivariable logistic regression analyses were performed to evaluate the independent association between mean lung density and in-hospital major adverse cardiovascular events (MACEs).

**Results:**

Two distinct subgroups were identified. The median (IQR) mean lung density was −806 [−826 to −785] HU in subgroup 1 and −727 [−747 to −704] HU in subgroup 2 (*P* < 0.001). Compared with subgroup 1, patients in the higher mean lung density phenotype (subgroup 2) exhibited significantly higher levels of cardiac enzymes, increased neutrophil and monocyte counts, and worse left ventricular function. Feature importance analysis identified markers of myocardial injury as the strongest correlates of increased mean lung density, followed by inflammatory cell counts and cardiac functional parameters. During hospitalization, 56 patients (20.3%) experienced MACEs, with a higher incidence in subgroup 2 than in subgroup 1 (30.4% vs. 14.4%). Increased mean lung density was associated with in-hospital MACEs in univariable analysis (OR per 1-SD increment, 1.92; 95% CI, 1.41–2.62; *P* < 0.001) and remained independently associated after adjustment for CK, LVEF, and neutrophil count (adjusted OR, 1.75; 95% CI, 1.24–2.47; *P* = 0.002).

**Conclusion:**

The higher mean lung density phenotype on CT, suggestive of subclinical pulmonary congestion, is associated with greater myocardial injury, heightened inflammatory activation, and impaired left ventricular function in patients with acute STEMI without overt HF. Multivariable analysis showed an independent association between increased mean lung density and in-hospital MACEs, supporting its potential role in early risk stratification among Killip class I patients.

## Introduction

Pulmonary congestion is a common complication in patients with acute myocardial infarction (MI) and is closely associated with heart failure (HF), hemodynamic deterioration, and adverse outcomes (1–4). However, pulmonary fluid accumulation may begin before overt symptoms or radiographic signs of HF become clinically apparent. Increasing evidence suggests that patients classified as Killip class I may already exhibit subtle increases in pulmonary fluid content despite the absence of clinically recognizable HF (5–7). This condition, often referred to as subclinical pulmonary congestion, may represent an early stage of cardiopulmonary injury after acute MI. Nevertheless, the clinical correlates and prognostic implications of subclinical pulmonary congestion in ST-segment elevation MI (STEMI) patients without overt HF remain incompletely understood.

Although chest radiography and lung ultrasound are useful for the rapid qualitative or semiquantitative assessment of pulmonary congestion in patients with acute MI (8, 9), both modalities exhibit limited sensitivity for detecting mild pulmonary congestion at the subclinical stage. Quantitative chest computed tomography (CT) provides an objective and highly sensitive method for assessing pulmonary tissue density and has been shown to correlate closely with pulmonary fluid content (10–12). More recently, Jain et al. demonstrated that quantitative CT can identify subclinical pulmonary congestion in patients with heart failure with preserved ejection fraction, revealing abnormalities that were not evident on conventional clinical evaluation (13). These findings suggest that CT-derived lung density may serve as a sensitive imaging biomarker for detecting early pulmonary congestion. However, whether quantitative CT can identify clinically meaningful subclinical pulmonary congestion in STEMI patients without overt HF has not been systematically investigated.

The mechanisms underlying pulmonary congestion after acute MI are complex and incompletely understood. Previous studies have shown that pulmonary congestion is caused by increased hydrostatic pressure due to left ventricular dysfunction and/or mitral regurgitation after acute MI (14–16). However, accumulating experimental and clinical evidence suggests that inflammatory processes may also play a crucial role. Acute MI triggers a robust systemic inflammatory response characterized by activation of circulating leukocytes, cytokine release, and endothelial dysfunction (17). Experimental studies have further demonstrated that acute MI can induce pulmonary vascular inflammation, increased endothelial permeability, and lung tissue injury independent of overt HF (18). Therefore, subclinical pulmonary congestion may represent an integrated manifestation of myocardial dysfunction and inflammatory activation rather than a consequence of any single factor.

Accordingly, we hypothesized that CT-derived mean lung density can identify subclinical pulmonary congestion in STEMI patients without overt HF and that such pulmonary congestion reflects the severity of myocardial injury and systemic inflammatory activation. The aims of this study were therefore to (1) characterize the clinical correlates of CT-detected subclinical pulmonary congestion in patients with acute STEMI without overt HF; and (2) evaluate its association with short-term adverse cardiovascular outcomes during hospitalization.

## Materials and methods

### Study population

Consecutive patients with acute STEMI without overt HF symptoms (Killip class I) who underwent chest CT without contrast enhancement between February 2015 and June 2021 were retrospectively enrolled. Inclusion criteria were acute STEMI with Killip class I presentation and availability of both chest CT and echocardiography on admission. The exclusion criteria were as follows: a history of previous MI or coronary revascularization; a history of pulmonary surgery; and patients with diseases that significantly alter lung density, such as lung tumors, emphysema, pulmonary tuberculosis, interstitial fibrosis, congenital lung abnormalities, and vascular disorders affecting pulmonary perfusion (e.g., chronic thromboembolism). Due to the retrospective nature of the study, the Ethics Committee of Chest Hospital, Tianjin University waived the requirement for written informed consent. All methods were performed in accordance with the relevant guidelines and regulations.

### Chest CT acquisition protocol

Chest CT scans were acquired using two CT scanners: a 256-slice CT scanner (Philips iCT, Philips Healthcare, Best, The Netherlands) and a 16-slice CT scanner (Emotion, Siemens Healthcare, Erlangen, Germany). All scans were performed in the supine position during end-inspiratory breath-hold, covering the entire lungs from the apex to the base. The scanning parameters were set as follows: tube voltage, 120 kV; automatic tube current modulation; image matrix, 512 × 512; reconstructed slice thickness, 1.5 mm; reconstruction kernel, L kernel for the Philips iCT scanner and B60s for the Siemens Emotion scanner; pitch, 1.0. The detector collimation was 128 × 0.625 mm for the Philips iCT scanner and 16 × 1.5 mm for the Siemens Emotion scanner. All CT scans were acquired without intravenous contrast enhancement.

The median time interval from symptom onset to CT acquisition was 5.1 h [interquartile range (IQR): 3.3–6.9 h], and the median time from CT acquisition to primary percutaneous coronary intervention (PCI) was 1.0 h (IQR: 0.9–1.5 h). The median door-to-balloon time in this cohort was 82 min (IQR: 71–94 min), which complied with the guideline-recommended 90-minute reperfusion target. Blood sampling for all laboratory tests was performed immediately upon hospital admission, prior to chest CT acquisition. No intravenous fluids or diuretics were administered to any patient before the CT examination.

### Clinical characteristics

Baseline demographic data (age, sex), vital signs (heart rate, respiratory rate), cardiovascular risk factors (hypertension, diabetes, smoking, dyslipidemia), laboratory biomarkers–including myocardial injury markers [creatine kinase (CK), CK-MB isoenzyme (CK-MB), lactate dehydrogenase (LDH), hydroxybutyrate dehydrogenase (HBDH), aspartate aminotransferase (AST), high-sensitivity cardiac troponin T (hs-cTnT)], inflammatory marker [high-sensitivity C-reactive protein (hs-CRP)], N-terminal pro-B-type natriuretic peptide (NT-proBNP), and white blood cell (WBC) counts [neutrophil, eosinophil, basophil, lymphocyte, monocyte]–along with echocardiographic parameters [left ventricular ejection fraction (LVEF), left ventricular end-diastolic diameter (LVEDD), left atrial anteroposterior diameter (LAAPD)] were systematically collected via electronic medical records review. Coronary angiography findings, including the presence of significant stenosis (≥ 50% diameter narrowing) in the left main (LM), left anterior descending (LAD), left circumflex (LCx), and right coronary artery (RCA), as well as the number of diseased vessels (1-vessel, 2-vessel, or 3-vessel disease), were also collected.

### Determination of the mean lung density

The chest CT images were transferred to the IntelliSpace Portal workstation (version 9.0, Philips Healthcare, Best, Netherlands) for postprocessing using the chronic obstructive pulmonary disease analysis tool. Based on a density threshold algorithm with an attenuation range of −1,000 HU (air) to 0 HU (water), the software automatically segmented the bilateral lung parenchyma and excluded non-parenchymal structures including the trachea, central bronchi, intrapulmonary vessels and mediastinal tissues. Mean lung density was calculated as the arithmetic mean of Hounsfield unit values across all valid parenchymal voxels, and total lung volume was recorded simultaneously. Two experienced radiologists (M.H. with 9 years of experience and J.S. with 6 years of experience) independently reviewed all segmentation masks and performed manual contour correction for inaccurate boundary segmentation; all observers were blinded to clinical data and patient outcomes. Representative examples of the segmentation procedure are provided in Supplementary Figure S1. Intra- and inter-observer reproducibility of mean lung density measurements was assessed using the intraclass correlation coefficient (ICC) in a randomly selected subset of 50 patients.

### Patient subgroups

Unsupervised learning evaluation metrics-the sum of squared errors (SSE) and silhouette coefficient (SC)-were used to determine the optimal number of subgroups based on the mean lung density in patients. The elbow point in the SSE curve and maximum value in the SC curve indicated the optimal cluster numbers (19). These two methods were performed using Python (Version 3.9.7). Patients were then clustered into distinct subgroups by the K-means algorithm (20) using the optimal cluster number. K-means clustering analysis was performed using IBM SPSS Statistics 26 (IBM Corporation, Armonk, NY, USA).

### Machine learning

A total of 24 clinical characteristics, with mean lung density and total lung volume excluded *a priori*, were used to develop a logistic regression model for discriminating the K-means-derived patient subgroups. First, all patients were randomly split into training (80%) and validation (20%) sets. Then, 10-fold cross-validation was used to optimize model parameters in the training set. Further, receiver operating characteristic (ROC) curves were used to evaluate the discriminative performance of the model based on the areas under the curves (AUCs). Finally, the clinical characteristics were ranked according to their importance for discriminating the patient subgroups.

### In-hospital follow-up

The primary in-hospital outcome of this study was the occurrence of major adverse cardiovascular events (MACEs). MACEs include all-cause death, acute coronary syndrome (ACS), malignant arrhythmia (ventricular tachycardia, ventricular fibrillation, and third-degree atrioventricular block), acute HF, cardiogenic shock, or stroke.

### Statistical analysis

Statistical analysis was performed using IBM SPSS Statistics 26. The one-sample Kolmogorov–Smirnov test was used to confirm a normal distribution. Normally distributed continuous variables were expressed as means ± standard deviations, whereas other variables were expressed as medians and IQRs. Categorical variables were expressed as counts (percentages). Student's *t*-test and Mann–Whitney *U* test were used to compare continuous variables with normal and non-normal distribution, respectively. Chi-square test was used to compare categorical variables. Univariable logistic regression analyses were first performed to estimate the crude associations between all candidate variables and in-hospital MACEs. Multivariable logistic regression was then constructed with in-hospital MACEs as the dependent variable. Covariates were selected based on *a priori* pathophysiological rationale rather than solely on univariable *P*-values. Mean lung density was analyzed both as a continuous variable (per 1-standard deviation increment) and as a K-means-derived phenotype to evaluate its independent association with in-hospital MACEs. A two-sided *P* value of < 0.05 was considered to indicate statistical significance.

## Results

### Patient characteristics

**Figure 1:**
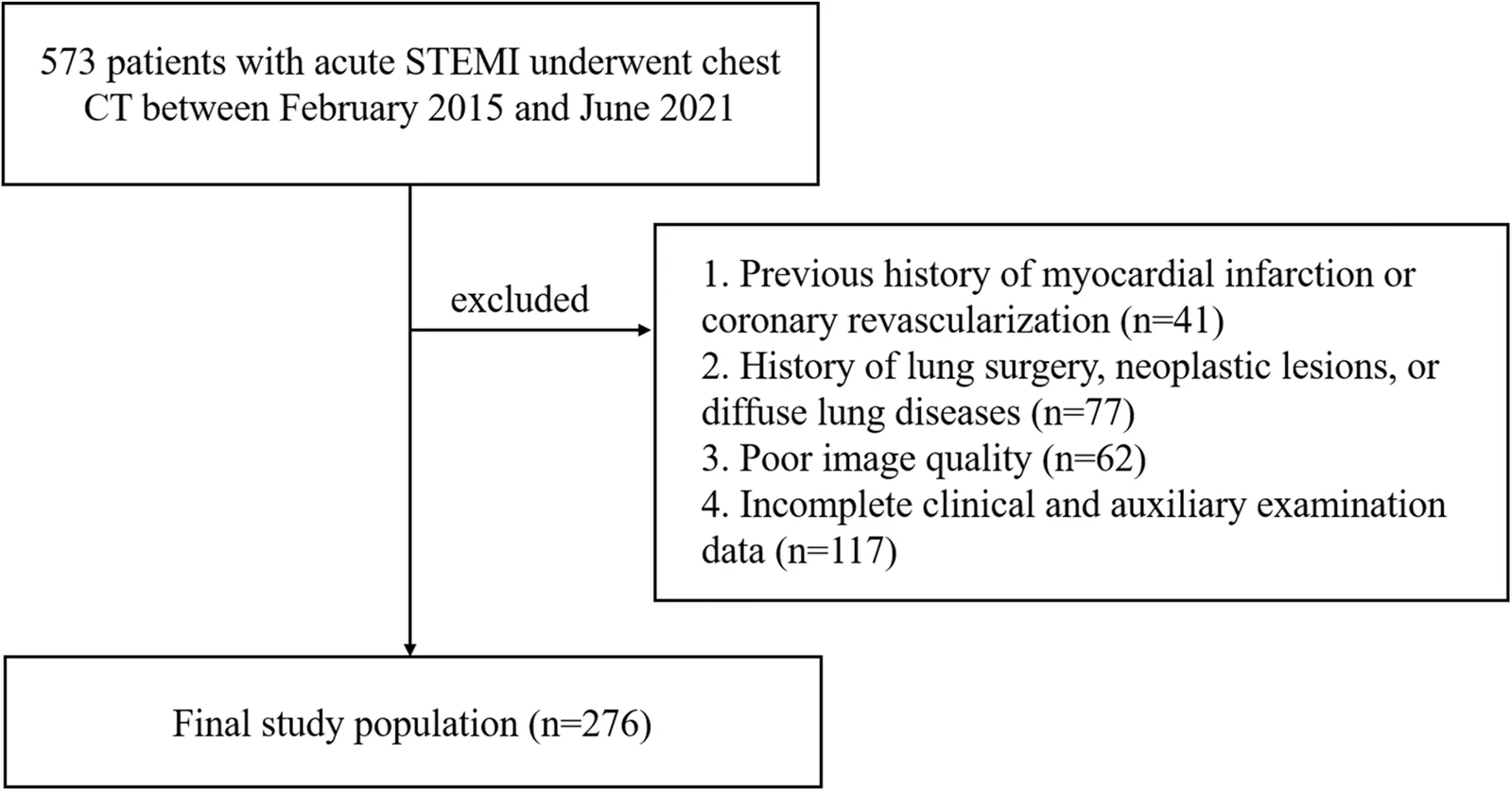
Flowchart of inclusion and exclusion criteria of the study. STEMI, ST-segment elevation myocardial infarction; CT, computed tomography.

Among 573 screened patients, 297 patients were excluded, as shown in Figure 1. Accordingly, the final population comprised 276 patients [221 men and 55 women; median age, 60 (IQR, 52, 67) years]. Patient clinical characteristics are presented in Table 1.

**Table 1:** Clinical characteristics of the study population.

Characteristic	Overall (*n* = 276)	Subgroup 1 (*n* = 174)	Subgroup 2 (*n* = 102)	*P* value
Age, y	60 (52, 67)	61 (53, 67)	58.5 (50, 68)	0.332
Male	221 (80)	146 (84)	75 (74)	0.037
Total lung volume, L	4.29 (3.68, 5.02)	4.32 (3.71, 5.05)	4.24 (3.62, 4.96)	0.482
Mean lung density, HU	−782 (−815, −744)	−806 (−826, −785)	−727 (−747, −704)	<0.001
Heart rate, beats/min	70 (63, 80)	70 (64, 80)	71 (60, 83)	0.656
Respiratory rate, breaths/min	16 (15, 18)	16 (15, 17)	16 (15, 18)	0.540
Hypertension	159 (58)	101 (58)	58 (57)	0.848
Diabetes	80 (29)	48 (28)	32 (31)	0.503
Dyslipidemia	118 (43)	77 (44)	41 (40)	0.511
Smoking	191 (69)	118 (68)	73 (72)	0.515
Laboratory biomarkers				
CK, U/L	1,325 (598, 2,730)	1,123 (526, 2,241)	2,183 (1,010, 3,355)	<0.001
CK-MB, U/L	144 (66, 250)	119 (58, 211)	205 (106, 346)	<0.001
LDH, U/L	539 (336, 853)	464 (289, 777)	749 (393, 1,059)	<0.001
HBDH, U/L	497 (282, 862)	419 (237, 783)	694 (346, 1,023)	<0.001
AST, U/L	152 (77, 267)	118 (64, 237)	225 (119, 333)	<0.001
hs-CRP, mg/L	5.86 (2.81, 12.39)	5.66 (2.28, 12.10)	6.62 (3.62, 13.01)	0.133
hs-cTnT, ng/mL	4.01 (1.71, 9.24)	3.71 (1.33, 7.70)	5.46 (2.79, 10.00)	0.003
NT-proBNP, pg/mL	716 (313, 1,572)	676 (292, 1,381)	954 (400, 1,708)	0.067
WBC count, 10^9^/L				
Neutrophil	7.45 (5.93, 9.67)	7.09 (5.54, 8.90)	7.96 (6.45, 10.55)	<0.001
Eosinophil	0.034 (0.009, 0.091)	0.047 (0.013, 0.102)	0.021 (0, 0.071)	0.003
Basophil	0.030 (0.021, 0.042)	0.029 (0.020, 0.042)	0.031 (0.022, 0.042)	0.272
Lymphocyte	1.50 (1.09, 2.00)	1.50 (1.10, 1.99)	1.54 (1.09, 2.15)	0.371
Monocyte	0.52 (0.40, 0.67)	0.50 (0.37, 0.64)	0.56 (0.44, 0.76)	0.007
Echocardiography				
LVEF, %	50.0 (46.2, 56.4)	50.1 (46.5, 57.3)	49.5 (45.8, 55.1)	0.030
LVEDD, mm	52 (49, 55)	51 (48, 54)	53 (49, 55)	0.047
LAAPD, mm	37 (34, 40)	38 (34, 40)	37 (34, 40)	0.540

Values are mean ± SD, *n* (%), or median (1st quartile, 3rd quartile). CK, creatine kinase, LDH, lactate dehydrogenase, HBDH, hydroxybutyrate dehydrogenase, AST, aspartate aminotransferase, hs-CRP, high-sensitivity C-reactive protein, hs-cTnT, high-sensitivity cardiac troponin T, NT-proBNP, N-terminal pro-B-type natriuretic peptide, WBC, white blood cell, LVEF, left ventricular ejection fraction, LVEDD, left ventricular end-diastolic diameter, LAAPD, left atrium anteroposterior diameter.

### Subgrouping and intersubgroup differences in clinical characteristics

**Figure 2:**
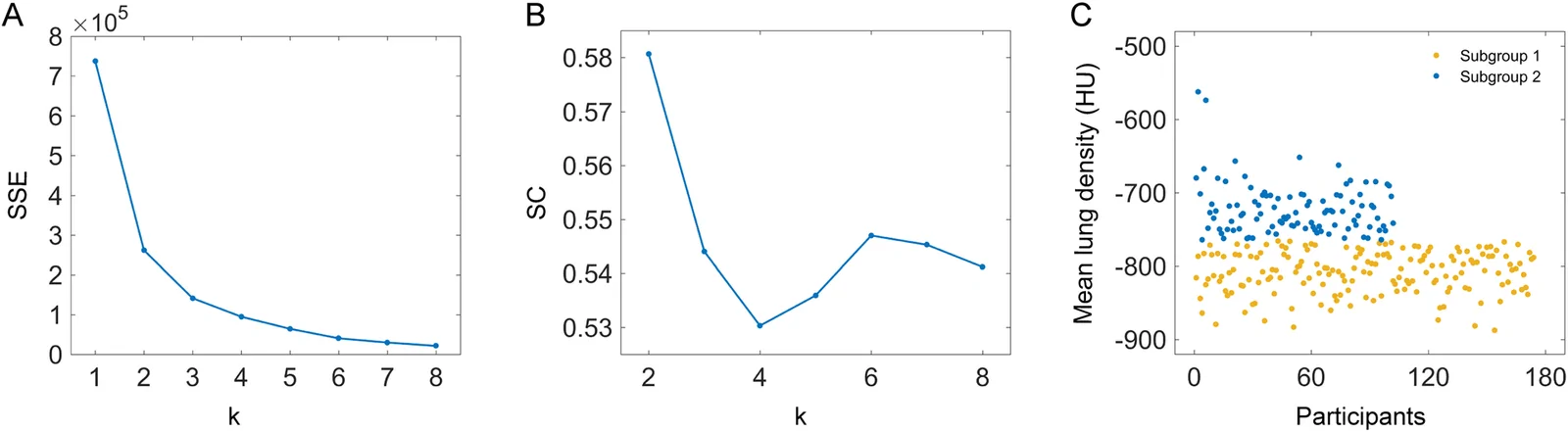
The sum of squared errors (SSE) and silhouette coefficient (SC) were used to determine the optimal number of subgroups based on the mean lung density **(A,B)**. The optimal number of patient subgroups was determined as two based on these two methods. The two patient subgroups were classified using K-means clustering analysis based on the mean lung density **(C)** k = number of subgroups.

**Figure 3:**
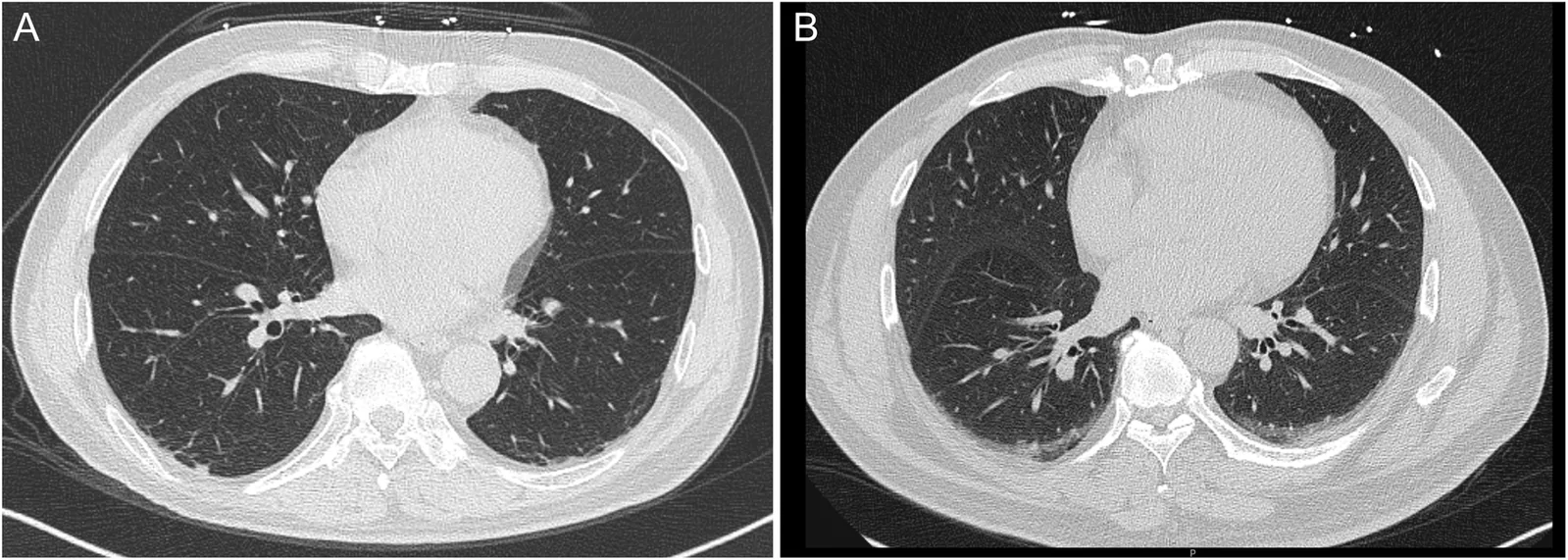
Examples of patients with STEMI in different subgroups. **(A)** CT image shows bilateral subpleural lines in the basal and posterior lung zones in a 60-year-old man in subgroup 1. **(B)** CT image shows bilateral subpleural band-like ground-glass opacities in the basal and posterior lung zones in a 52-year-old man in subgroup 2. STEMI = ST-segment elevation myocardial infarction.

As shown in Figures 2A,B, the optimal cluster number was determined as two based on both SSE and SC curves. Overall, 174 (63%) and 102 (37%) patients were assigned to subgroup 1 and 2, respectively, using K-means clustering analysis based on their mean lung density. There were more men in subgroup 1 than in subgroup 2 (84% vs. 74%, *P* = 0.037). Subgroup 2 had higher mean lung density than subgroup 1 [median (IQR), −727 (−747, −704) vs. −806 (−826, −785) HU, *P* < 0.001). Total lung volume did not differ significantly between the two subgroups (median [IQR], 4.32 [3.71, 5.05] vs. 4.24 [3.62, 4.96] L, *P* = 0.482), suggesting that the observed difference in mean lung density was unlikely attributable to differences in inspiratory effort. The distribution of both subgroups is presented in Figure 2C. On chest CT images, subgroup 1 mainly manifests as subpleural lines and/or slight interlobular septal thickening, or an absence of overt abnormalities. In contrast, subgroup 2 mainly manifests as subpleural band-like ground-glass opacities, mild interlobular thickening, and/or faint small patchy shadows. Representative examples from each subgroup are provided in Figure 3. The distribution of the two CT scanners was balanced between subgroups. Overall, 208 patients (75.4%) were scanned on the Philips iCT scanner, with comparable proportions in subgroup 1 (75.9%) and subgroup 2 (74.5%; *P* = 0.792).

There were no significant differences in age, vital signs (heart rate, respiratory rate), and cardiovascular risk factors (hypertension, diabetes, smoking, dyslipidemia) between the subgroups, all *P* > 0.05. Based on laboratory biomarkers, subgroup 2 showed significantly higher levels of CK, CK-MB, LDH, HBDH, AST and hs-cTnT, higher counts of neutrophils and monocytes, and a lower eosinophil count than subgroup 1, all *P* < 0.05. Echocardiography revealed that subgroup 2 had significantly lower LVEF and higher LVEDD than subgroup 1. Detailed clinical characteristics of the two subgroups are summarized in Table 1. As shown in Table 2, there were no significant differences in the prevalence of multivessel disease, specific coronary artery involvement, or the distribution of infarct-related (culprit) arteries between the two subgroups (all *P* > 0.05).

**Table 2:** Angiography findings of the Two subgroups.

Angiography findings	Subgroup 1 (*n* = 174)	Subgroup 2 (*n* = 102)	*P* value
No significant disease	2 (1.1%)	1 (1.0%)	0.896
LM disease	7 (4.0%)	5 (4.9%)	0.730
LAD disease	149 (85.6%)	90 (88.2%)	0.540
LCx disease	99 (56.9%)	67 (65.7%)	0.150
RCA disease	113 (64.9%)	69 (67.6%)	0.647
1-vessel disease	50 (28.7%)	21 (20.6%)	0.135
2-vessel disease	55 (31.6%)	36 (35.3%)	0.530
3-vessel disease	67 (38.5%)	44 (43.1%)	0.449
Infarct-related artery			
LM	1 (0.6%)	0 (0.0%)	1.000
LAD	92 (52.9%)	53 (52.0%)	0.883
LCx	32 (18.4%)	21 (20.6%)	0.655
RCA	52 (29.9%)	37 (36.3%)	0.273

Values are mean ± SD or *n* (%). LM, left main coronary; LAD, left anterior descending artery; LCx = left circumflex coronary artery; RCA = right coronary artery.

### Machine learning for discriminating the subgroups

**Figure 4:**
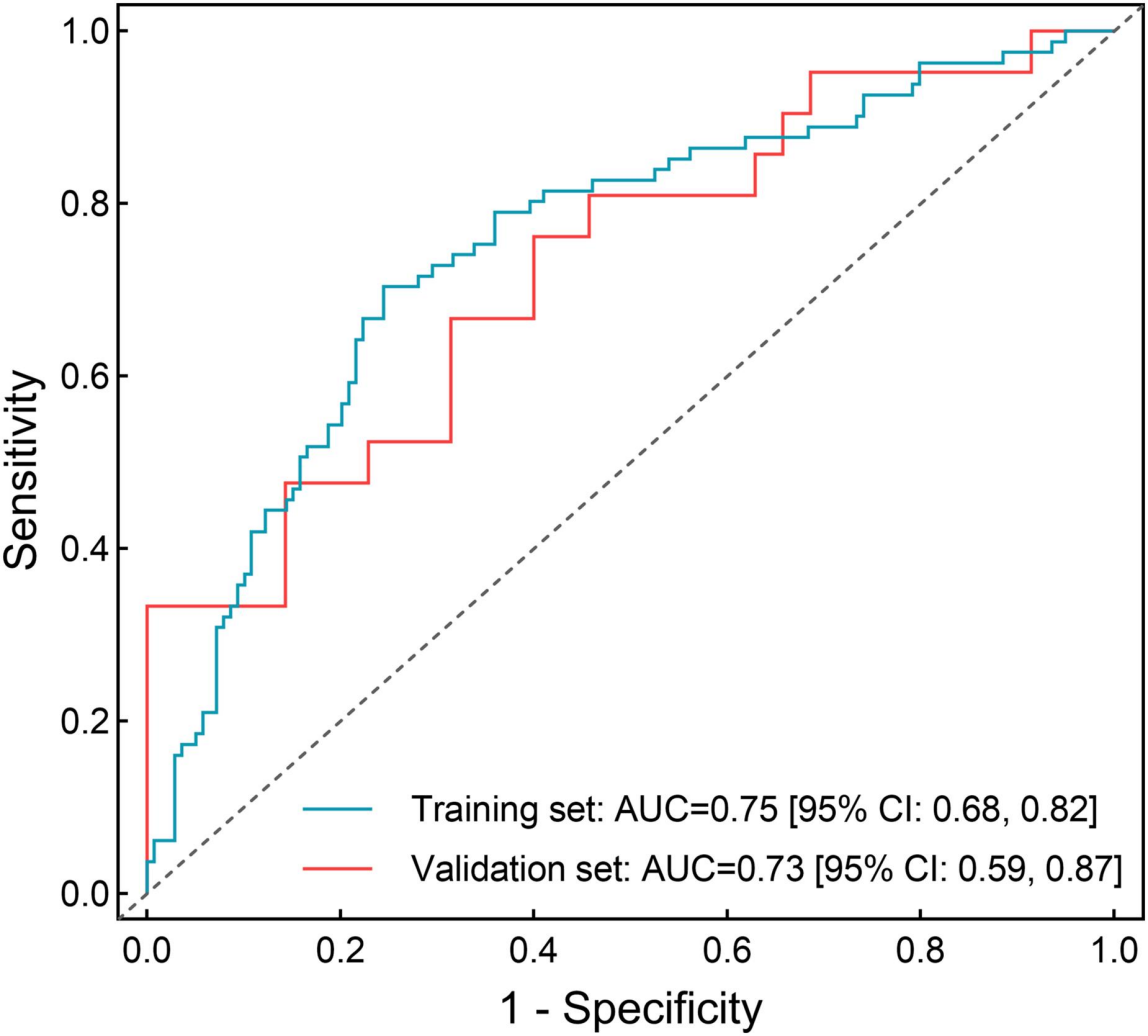
Performance of the logistic regression model in discriminating subgroups in the training and validation sets using 24 clinical characteristics. AUC=area under the curve.

**Figure 5:**
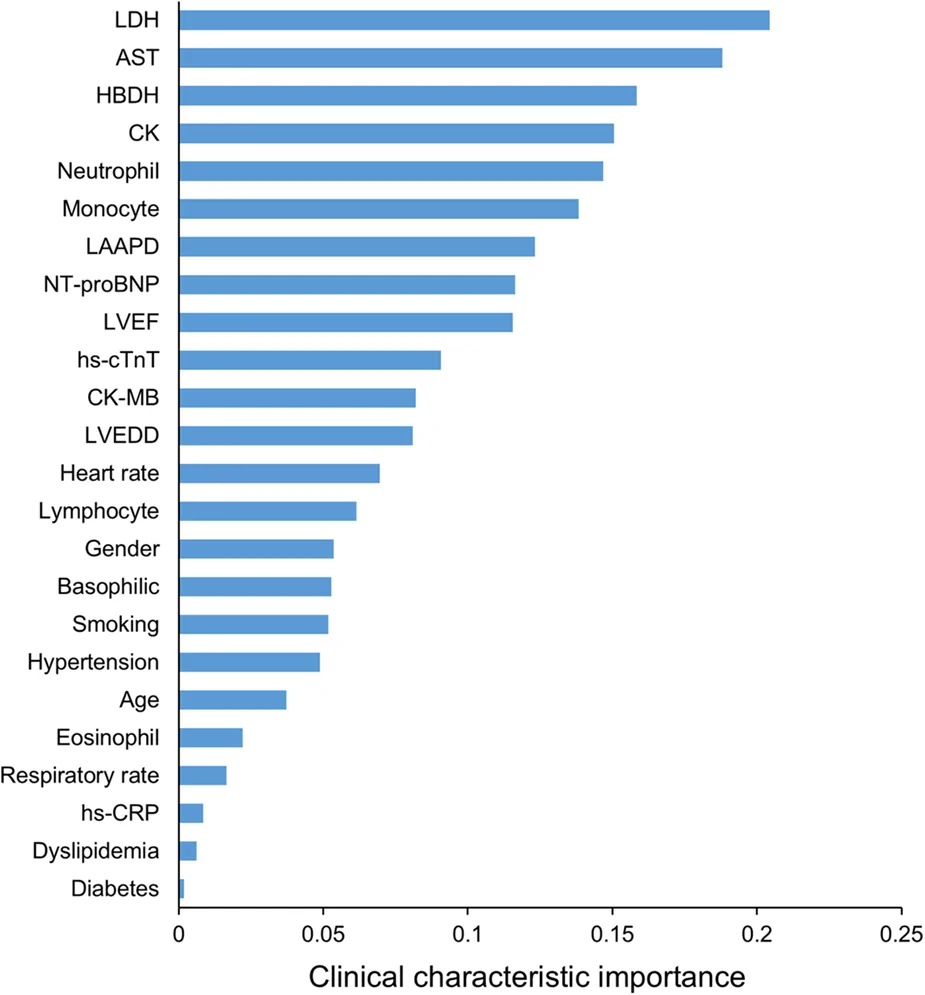
The ranking of 24 clinical characteristics, according to their importance, in the logistic regression model. CK, creatine kinase; LDH, lactate dehydrogenase; HBDH, hydroxybutyrate dehydrogenase; AST, aspartate aminotransferase; hs-CRP, high-sensitivity C-reactive protein; hs-cTnT, high-sensitivity cardiac troponin T; NT-proBNP, N-terminal pro-B-type natriuretic peptide; LVEF, left ventricular ejection fraction; LVEDD, left ventricular end-diastolic diameter; LAAPD, left atrium anteroposterior diameter.

A logistic regression model was developed for discriminating the subgroups based on 24 clinical characteristics. As shown in Figure 4, the model yielded an AUC of 0.75 (95% CI: 0.68–0.82) in the training set and 0.73 (95% CI: 0.59–0.87) in the validation set, indicating favorable discriminative performance for clinical application. Figure 5 displays the ranking of clinical characteristics by their predictive importance, which presented a distinct category-based hierarchy. Cardiac enzymes (LDH, AST, HBDH, CK) dominated the top four positions, followed by inflammatory cells (neutrophils, monocytes) and cardiac function parameters (LAAPD, NT-proBNP, LVEF). General clinical variables such as age, sex, and comorbidities contributed minimally.

### In-hospital outcome

In total, 56 patients (20.3%) experienced MACEs during hospitalization, including 24 acute HF cases, 17 malignant arrhythmias cases, 10 ACS cases, 4 cardiogenic shock cases, and 1 death. MACEs occurred in 25 patients (14.4%) in subgroup 1 and 31 patients (30.4%) in subgroup 2. Univariable analysis showed that higher mean lung density was associated with in-hospital MACEs, both as a continuous variable (OR 1.92 per 1-SD increase, 95% CI 1.41–2.62, *P* < 0.001; Table 3) and as the K-means-derived subgroup (OR 2.60, 95% CI 1.43–4.73, *P* = 0.002; Supplementary Table S1). After adjustment for CK, LVEF, and neutrophil count, mean lung density remained independently associated with in-hospital MACEs (adjusted OR 1.75, 95% CI 1.24–2.47, *P* = 0.002; Table 3). Similar results were obtained when mean lung density was entered as the subgroup variable (adjusted OR 2.33, 95% CI 1.21–4.49, *P* = 0.011; Supplementary Table S2). Additional adjustment for scanner type did not materially alter these associations (Supplementary Table S2). Univariable logistic regression results for all candidate variables are presented in Supplementary Table S1.

**Table 3:** Logistic regression analysis for the prediction of in-hospital MACEs.

	Univariable analysis		Multivariable analysis	
Variable	OR (95% CI)	*P* value	OR (95% CI)	*P* value
Mean lung density, HU	1.92 (1.41–2.62)	<0.001	1.75 (1.24–2.47)	0.002
CK, U/L	1.54 (1.17–2.01)	0.002	0.93 (0.67–1.29)	0.668
LVEF, %	0.44 (0.32–0.61)	<0.001	0.49 (0.35–0.70)	<0.001
Neutrophil, 10^9^/L	1.82 (1.30–2.55)	<0.001	1.59 (1.09–2.33)	0.017

CK, creatine kinase, LVEF, left ventricular ejection fraction, CI, confidence interval, OR, odds ratio.

### Reproducibility of mean lung density measurements

The reproducibility of mean lung density measurement was excellent, with an intra-observer ICC of 0.97 (95% CI: 0.95–0.99) and an inter-observer ICC of 0.95 (95% CI: 0.91–0.97) in the randomly selected subset of 50 patients.

## Discussion

The present study investigated the clinical correlates and prognostic value of a higher mean lung density phenotype on CT, which is suggestive of subclinical pulmonary congestion, in patients with acute STEMI without overt HF. Using quantitative mean lung density measurements and unsupervised clustering analysis, we identified two distinct patient phenotypes characterized by different degrees of pulmonary congestion. Our findings showed (1): patients with higher mean lung density exhibited significantly greater myocardial injury, increased inflammatory cell counts, and impaired left ventricular function (2); machine learning analysis demonstrated that markers of myocardial injury, inflammatory activation, and cardiac dysfunction were the dominant determinants associated with subclinical pulmonary congestion; (3) increased mean lung density was independently associated with in-hospital MACEs after adjustment for markers of myocardial injury (CK), left ventricular function (LVEF), and inflammation (neutrophil count). Collectively, these findings suggest that subclinical pulmonary congestion represents an early manifestation of cardiopulmonary injury after STEMI and provides important prognostic information.

### Quantitative CT enables early detection of subclinical pulmonary congestion

Pulmonary congestion has traditionally been considered a consequence of overt HF following acute MI. However, growing evidence suggests that pulmonary fluid accumulation may occur before clinical signs or symptoms become apparent. Jain et al. showed that quantitative chest CT could identify subclinical pulmonary congestion in patients with heart failure with preserved ejection fraction, even in the absence of overt decompensation (13). Moreover, Barile et al. demonstrated that quantitative lung CT outperforms chest X-ray and visual reading in detecting pulmonary congestion, enabling detection of subclinical fluid accumulation at an early stage (10). These findings suggest that CT may be highly sensitive in detecting early pulmonary congestion before the development of radiographically evident edema or clinically significant respiratory symptoms. In this study, our findings are consistent with previous studies and demonstrate that quantitative CT enables early detection of subclinical pulmonary congestion in patients with acute STEMI without overt HF. All scans were acquired during standardized full-inspiration breath-hold, and patients with overt parenchymal lung diseases that alter lung density were excluded at study entry, which largely mitigates but does not fully eliminate confounding from mild dependent atelectasis or subclinical inflammatory changes.

### Potential mechanisms underlying subclinical pulmonary congestion after STEMI

Our study identified cardiac enzymes (LDH, AST, HBDH, and CK) as the most important predictors of subclinical pulmonary congestion. This finding aligns with previous evidence demonstrating that total CK and LDH levels correlate more closely with infarct size than troponin isoforms in the early phase of STEMI (21, 22). Nienhuis et al. (21) reported that peak CK levels after primary PCI were independently associated with left ventricular dysfunction and long-term mortality in STEMI patients. Similarly, Turer et al. (22) showed that enzyme estimates of infarct size correlated better with global left ventricular function than troponin levels. The stronger association between non-specific cardiac enzymes and subclinical pulmonary congestion likely reflects the fact that these markers integrate the total burden of myocardial injury, which is the fundamental determinant of hemodynamic compromise and subsequent pulmonary fluid accumulation.

The classic pathophysiological mechanism of pulmonary congestion after MI involves increased hydrostatic pressure secondary to left ventricular dysfunction, which elevates pulmonary capillary hydrostatic pressure and drives fluid extravasation into the interstitium (15, 16). In our study, patients with subclinical pulmonary congestion had significantly lower LVEF and higher LVEDD compared to those without, supporting this hemodynamic mechanism. However, it is noteworthy that the absolute difference in LVEF and LVEDD between the two subgroups was relatively small, suggesting that impaired systolic function alone cannot fully explain the observed increase in mean lung density. This is further supported by the lack of significant difference in coronary angiographic findings between the two subgroups, indicating that subclinical pulmonary congestion is not simply a consequence of more extensive coronary artery disease but rather reflects the individual response to myocardial injury.

Beyond hemodynamic alterations, inflammation may also contribute to pulmonary congestion formation. Patients with increased lung density demonstrated significantly higher neutrophil and monocyte counts, and inflammatory cell counts ranked among the strongest predictors in the machine learning model. Acute MI is known to trigger systemic inflammatory responses characterized by leukocyte activation, cytokine release, and recruitment of inflammatory cells to injured tissues (23, 24). Further studies have shown that MI can induce pulmonary endothelial activation, increased vascular permeability, and inflammatory cell infiltration within the lungs (17, 25). These changes may facilitate pulmonary fluid accumulation independently of hydrostatic pressure.

Taken together, our findings demonstrate that subclinical pulmonary congestion following STEMI is not merely a consequence of elevated cardiac filling pressures but rather reflects the combined effects of myocardial injury, inflammatory activation, and impaired cardiac function (4). Therefore, CT-detected subclinical pulmonary congestion may serve as an integrated imaging marker reflecting the overall severity of cardiopulmonary injury during the acute phase of STEMI.

### Prognostic significance of subclinical pulmonary congestion

The most clinically relevant finding of our study is the independent association between subclinical pulmonary congestion and in-hospital outcomes. Increased mean lung density was associated with in-hospital MACEs in univariable analysis, and the association remained significant after adjustment for CK, LVEF, and neutrophil count. Consistent results were obtained when mean lung density was analyzed as the K-means-derived subgroup.

Our results extend previous findings on the prognostic importance of pulmonary congestion in acute MI. While chest radiography and lung ultrasound have been shown to predict adverse outcomes in STEMI patients (8, 9), these modalities are relatively insensitive to mild interstitial edema. We observed that quantitative CT could identify a much earlier stage of pulmonary congestion before overt HF develops. Importantly, all patients in our cohort were classified as Killip class I and therefore would traditionally be considered relatively low risk. Nevertheless, patients with increased mean lung density experienced significantly worse outcomes. This finding suggests that CT-derived subclinical pulmonary congestion may uncover hidden risk not apparent through conventional clinical evaluation. Therefore, early identification of subclinical pulmonary congestion may facilitate more accurate risk stratification and individualized management.

### Limitations

There are several limitations in our study. First, this was a retrospective single-center study restricted to STEMI patients who underwent clinically indicated chest CT, accounting for only approximately 5.5% of all STEMI patients receiving primary PCI during the study period. This highly selected cohort introduces inherent selection bias, and our findings may not be fully generalizable to unselected Killip class I STEMI patients. Second, chest CT examinations were performed at a single time point, preventing assessment of temporal changes in pulmonary congestion during hospitalization. Third, chest CT examinations were performed on two different scanners, which may introduce inter-scanner systematic bias in the quantification of mean lung density. Nevertheless, additional adjustment for scanner type in the multivariable models did not materially change the association between mean lung density and MACEs. Fourth, direct measurements of pulmonary capillary wedge pressure, extravascular lung water, or independent congestion assessments (e.g., lung ultrasound) were unavailable to validate the CT-derived mean lung density phenotype as a definitive marker of subclinical pulmonary congestion. Finally, only in-hospital outcomes were evaluated, and the long-term prognostic significance of CT-detected subclinical pulmonary congestion remains to be determined.

## Conclusions

Our findings demonstrate that the CT-detected higher mean lung density phenotype, suggestive of subclinical pulmonary congestion, is associated with greater myocardial injury, heightened inflammatory activation, and impaired left ventricular function in patients with acute STEMI without overt HF. Importantly, multivariable logistic regression analysis showed an independent association between increased mean lung density and in-hospital MACEs after adjustment for CK, LVEF, and neutrophil count, supporting its potential role in early risk stratification among patients clinically classified as Killip class I.

## Data Availability

The data analyzed in this study is subject to the following licenses/restrictions: The dataset contains sensitive clinical and imaging information of human participants. Public sharing is restricted by institutional ethical policies and patient privacy protection regulations. All de-identified data are available from the corresponding author upon reasonable request. Requests to access these datasets should be directed to HZ, tjch_zhanghong@163.com.
