# Supplementary material for: Clinical correlates and short-term prognostic value of CT-detected subclinical pulmonary congestion in acute STEMI without overt heart failure
Source: Front Med (Lausanne). 2026 Sep 8;13:1927125. doi: 10.3389/fmed.2026.1927125 (PMC13593481; doi:10.3389/fmed.2026.1927125)
Supplement: Supplementary file 1 [file Table1.docx]

**Clinical Correlates and Short-Term Prognostic Value of CT-Detected Subclinical Pulmonary Congestion in Acute STEMI Without Overt Heart Failure**

**Supplementary Table S1. Univariable logistic regression** **analysis for the prediction of** **in-hospital MACEs.**

| Variable | OR (95% CI) | P value |
| --- | --- | --- |
| Age, y | 1.30 (0.96–1.76) | 0.091 |
| Male | 0.61 (0.31–1.20) | 0.153 |
| Total lung volume, L | 1.17 (0.87–1.56) | 0.294 |
| Mean lung density, HU | 1.92 (1.41–2.62) | <0.001 |
| Mean lung density subgroup  (Subgroup 2 vs Subgroup 1) | 2.60 (1.43–4.73) | 0.002 |
| Siemens scanner vs Philips scanner | 0.52 (0.24–1.13) | 0.100 |
| Heart rate, beats/min | 0.85 (0.64–1.15) | 0.300 |
| Respiratory rate, breaths/min | 0.93 (0.69–1.25) | 0.610 |
| Hypertension | 0.89 (0.49–1.61) | 0.703 |
| Diabetes | 1.34 (0.72–2.51) | 0.362 |
| Dyslipidemia | 0.47 (0.11–2.12) | 0.327 |
| Smoking | 1.03 (0.54–1.94) | 0.936 |
| CK, U/L | 1.54 (1.17–2.01) | 0.002 |
| CK-MB, U/L | 1.47 (1.12–1.93) | 0.005 |
| LDH, U/L | 1.52 (1.15–2.00) | 0.003 |
| HBDH, U/L | 1.50 (1.14–1.98) | 0.004 |
| AST, U/L | 1.41 (1.08–1.85) | 0.013 |
| hs-CRP, mg/L | 1.43 (1.11–1.85) | 0.006 |
| hs-cTnT, ng/mL | 1.32 (0.99–1.76) | 0.055 |
| NT-proBNP, pg/mL | 1.95 (1.36–2.81) | <0.001 |
| Neutrophil, 10^9^/L | 1.82 (1.30–2.55) | <0.001 |
| Eosinophil, 10^9^/L | 0.27 (0.10–0.69) | 0.006 |
| Basophil, 10^9^/L | 0.75 (0.51–1.10) | 0.142 |
| Lymphocyte, 10^9^/L | 1.12 (0.84–1.49) | 0.460 |
| Monocyte, 10^9^/L | 0.94 (0.70–1.26) | 0.682 |
| LVEF, % | 0.44 (0.32–0.61) | <0.001 |
| LVEDD, mm | 1.41 (1.05–1.91) | 0.024 |
| LAAPD, mm | 1.15 (0.86–1.55) | 0.340 |

CK = creatine kinase, LDH = lactate dehydrogenase, HBDH = hydroxybutyrate dehydrogenase, AST = aspartate aminotransferase, hs-CRP = high-sensitivity C-reactive protein, hs-cTnT = high-sensitivity cardiac troponin T, NT-proBNP = N-terminal pro-B-type natriuretic peptide, LVEF = left ventricular ejection fraction, LVEDD = left ventricular end-diastolic diameter, LAAPD = left atrium anteroposterior diameter, CI = confidence interval, OR = odds ratio.

**Supplementary Table S2. Multivariable logistic regression analysis for the prediction of** **in-hospital MACEs.**

| Model | OR (95% CI) | P value |
| --- | --- | --- |
| Model 1 |  |  |
| Mean lung density, HU | 1.75 (1.24–2.47) | 0.002 |
| CK, U/L | 0.93 (0.67–1.29) | 0.668 |
| LVEF, % | 0.49 (0.35–0.70) | <0.001 |
| Neutrophil, 10^9^/L | 1.59 (1.09–2.33) | 0.017 |
| Model 2 |  |  |
| Mean lung density subgroup  (Subgroup 2 vs Subgroup 1) | 2.33 (1.21–4.49) | 0.011 |
| CK, U/L | 1.00 (0.73–1.38) | 0.989 |
| LVEF, % | 0.49 (0.34–0.69) | <0.001 |
| Neutrophil, 10^9^/L | 1.61 (1.10–2.36) | 0.015 |
| Model 3 |  |  |
| Mean lung density, HU | 1.77 (1.25–2.51) | 0.001 |
| CK, U/L | 0.90 (0.65–1.26) | 0.559 |
| LVEF, % | 0.48 (0.34–0.69) | <0.001 |
| Neutrophil, 10^9^/L | 1.57 (1.07–2.29) | 0.021 |
| Siemens scanner vs Philips scanner | 0.49 (0.21–1.12) | 0.091 |
| Model 4 |  |  |
| Mean lung density subgroup  (Subgroup 2 vs Subgroup 1) | 2.34 (1.21–4.54) | 0.012 |
| CK, U/L | 0.98 (0.71–1.35) | 0.905 |
| LVEF, % | 0.48 (0.33–0.68) | <0.001 |
| Neutrophil, 10^9^/L | 1.58 (1.08–2.32) | 0.018 |
| Siemens scanner vs Philips scanner | 0.50 (0.22–1.15) | 0.103 |

CK = creatine kinase, LVEF = left ventricular ejection fraction, CI = confidence interval, OR = odds ratio.

**Supplementary Figure S1** Representative images of the lung parenchyma segmentation procedure for CT-derived mean lung density quantification.
